# Facile tuning of tip sharpness on gold nanostars by the controlled seed-growth method and coating with a silver shell for detection of thiram using surface enhanced Raman spectroscopy (SERS)†

**DOI:** 10.1039/d2ra03396h

**Published:** 2022-08-15

**Authors:** Anh Thi Ngoc Quang, Thu Anh Nguyen, Sy Van Vu, Tien Nu Hoang Lo, In Park, Khuong Quoc Vo

**Affiliations:** Institute of Applied Technology, Thu Dau Mot University 6 Tran Van On Street, Phu Hoa Ward Thu Dau Mot City Binh Duong Province Vietnam; Faculty of Chemistry, Ho Chi Minh City University of Science, Vietnam National University 227 Nguyen Van Cu Street, Ward 4, District 5 Ho Chi Minh City 70000 Vietnam vqkhuong@hcmus.edu.vn; Research Institute of Clean Manufacturing System, Korea Institute of Industrial Technology (KITECH) 89 Yangdaegiro-gil, Ipjang-myeon Cheonan 31056 South Korea inpark@kitech.re.kr; KITECH School, University of Science and Technology (UST) 176 Gajeong-dong, Yuseong-gu Daejeon 34113 South Korea

## Abstract

Developing SERS substrates based on individual gold and silver metals, either with rough surfaces or bare nanoparticles, has certain limitations in practical analysis applications. In order to improve the range of applications of the noble metallic substrates, a comprehensive approach has been proposed for preparing non-traditional SERS nano-substrates by combining tip-enhanced gold nanostars and Raman signal amplification of the silver layer. This preparation process is conducted in two steps, including tuning the sharpness and length of tips by a modified seed growth method followed by coating the silver layer on the formed star-shaped nanoparticles. The obtained AuNS-Ag covered with an average size of around 100 nm exhibited interesting properties as a two-component nano-substrate to amplify the activities in SERS for detecting thiram. The controllable and convenient preparation route of gold nanostars is based on the comproportionation reaction of Au seed particles with Au(iii) ions, achieved by governing the stirring times of the mixture of the Au seed and the growth solution. Thus, the citrate-seed particles decreased in size (below 2 nm) and grew into nanostars with sharp tips. The thickness of Ag covering the Au particles' surface also was appropriately controlled and the tips were still exposed to the outside, which is a benefit for matching with the source excitation wavelength to achieve good SERS performance. The Raman signals of thiram can be instantly and remarkably detected with the enhancement of the substrates. Thiram can be determined without any pretreatment. It was found that the limit of detection for thiram is 0.22 ppm, and the limit of quantification is 0.73 ppm. These experimental results shed some light on developing the SERS method for detecting pesticide residue.

## Abstract 2

Developing SERS substrates based on the star-like morphology of gold nanoparticles covered by a silver layer to overcome limitations in practical analysis application.

## Graphical abstract

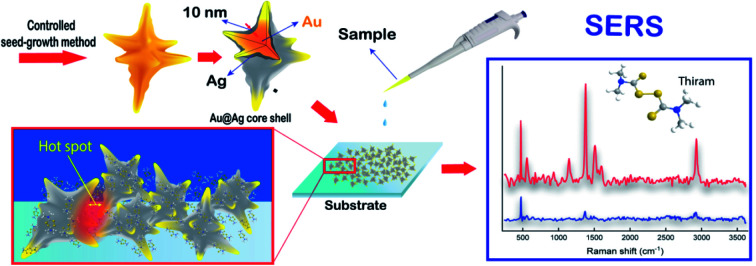

## Introduction

**Fig. 1:**
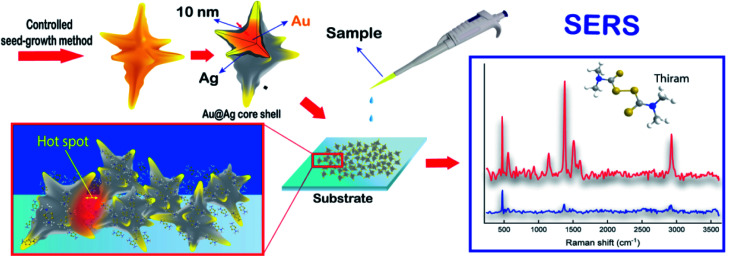
Preparation of AuNS@Ag substrate with controlled seed growth method and SERS detection of thiram molecules.

SERS is an ultrasensitive vibrational spectroscopic technique that can identify molecular traces with significant enhancement mechanization.^1–3^ In recent years, a substantial amount of work on SERS to determine chemical residues has been reported because of specific advantages of this spectroscopic method, including high sensitivity,^4,5^ selectivity,^6^ low interference,^7^ and easy *in situ* monitoring.^8,9^ The SERS signal intensity strongly depends on many factors, including the type of organic molecules, the number of molecules, and metal nano-substrate (such as material type, size distribution, and shape).^10^ Precious metallic nano-substrates such as gold, silver, and platinum have recently attracted significant attention due to their specific surface and morphologies. These are widely believed to be responsible for the electron density distribution; thus, this affects the electric field around the nanoparticles.^11–15^ The electromagnetic field gives rise to the Raman signal as the molecules adsorbed specifically in the “hot spots” or intense fields of localized surface plasmon.^16^ Among various kind of morphologies, spike nanostructures with unique plasmonic effects, such as gold nanostars,^11,17,18^ are ideal materials for designing efficient surface-enhanced Raman scattering (SERS) substrates.^19–21^ The star-like morphology possesses many sharp tips, minor grooves, and edges, where excitation light can be favorably concentrated.^22^ As excited under specific conditions, the gold nanostars can be generated a local large electromagnetic field near the sharp tips at the ends of their branches.^13,23^ The hybridization of the cores and tips induces a high localized surface plasmon resonances (LSPR) feature with an absorption band in the near-infrared (NIR) range (650–1100 nm) compared to spherical particles of identical size,^24^ leading to an enhancement in the local electric field.^25^ The LSPR of the star-like gold nanostructure is also close to the excitation wavelength of the Raman source (532–785 nm). These phenomenon could enhance the Raman scattering signal of the localized molecules on the surface of the nanoparticles by multiple orders of magnitude.^20^ In addition, developing multicomponent nanostructures have also become an important, challenging goal because of these material compositions and physicochemical properties.^26^ Due to the synergistic effects and easy fabrication in mild synthesis conditions, bimetallic Au@Ag nanostructures are increasingly studied for Raman signal performance.^27^ Of the plasmonic metals, silver nanoparticles contribute fascinating optical properties due to the strong surface plasmon across the spectrum from 300 to 1200 nm, while gold is well-known for its inertness and simple to tune in the particle morphologies.^28^ Employing a shell of silver enables the electromagnetic field to expand further from the surface, thus improving SERS performance than single solid particles (Fig. 1).^14^

As a protective fungicide, tetramethyl thiuram disulfide (thiram) is extensively employed in agriculture to protect crop cultivation. Prolonged exposure with high levels of thiram can cause hand eczema or dermatitis,^29^ severe eye irritation,^30^ slight erythema,^30^ possibly vomiting, headaches, and nausea.^8^ Although conventional analysis methods for detecting thiram such as high-performance liquid chromatography (HPLC)-electrochemical detection,^31^ gas chromatography,^32^ and colorimetric method^33^ can achieve good determination results involving high selectivity, reproductivity, and sensitivity. Their disadvantages in actual applications are complicated, time-consuming, and expensive sample pretreatment. As an optical strategy with the advantages of simplicity, minor sample preparation, and high sensitivity, surface-enhanced Raman spectroscopy (SERS) are of significant interest for sensing application. Various nano-substrates have been currently designed and synthesized to determine thiram. Hussain *et al.* have developed the bimetallic core–shelled Au@Ag nanoparticles to determine thiram contaminants in milk through SERS.^4^ Li *et al.* fabricated single crystal silver nanowires as the SERS substrates with many advantages, including simplicity and a large amount of synthesis for determining the pesticide thiram. The detection limit was 1 × 10^−7^ M with a low RSD of 20%.^34^ Yuan *et al.* synthesized self-assembled silver nanoclusters with dense multiple active sites and hot spots for the high-performance SERS detection of thiram. The single cluster produced a much low detection limit of 0.024 ppm.^35^ Zhang *et al.* developed the novel Au@AgNPs/GO/Au@AgNPs sandwich nanostructure film, which can detect thiram in natural lake water and commercial grape juice. This nanostructure with many hot spots on the surface can significantly enhance the Raman signal of thiram with a detection limit of 0.1 μM (0.03 ppm).^36^ Zhu *et al.* reported the synthesis of multibranched gold nanostars with fractal structures. The experimental results showed that this substrate could produce a stronger signal than gold nanostars without fractal features with a detection limit as low as 10^−10^ M in solution and 0.24 ng cm^−2^ in apple peels.^37^

Though a large amount of work was proposed to prepare substrates for thiram SERS detection based on gold and silver nanoparticles with different techniques, surfactants, or reducing agents, fewer studies on the two components substrate with the Au core existed as star-liked morphology and covered by the silver layers. One aspect of this research focuses on the easy tuning of the branches' length on the star-liked nanoparticles through investigating the con–proportionate reaction of Au seed nanoparticles with Au^3+^ ions conducted before aging; without using additive agents or finding a new mixture of surfactants. The gold seed nanoparticles were allowed to grow by adding a reducing agent after an appropriate con–proportionate reaction interval for obtaining well-formed star-liked morphology. The star-liked gold nanoparticles were further experience in a simple coating process with the silver layer on the surface of gold nanoparticles depending on the addition of AgNO_3_ concentration. A major advantage of covering Au nanostars with the Ag layer is combining the plasmonic effect of the branches on star-like gold nanoparticles and the optical enhancement property of Ag. Specifically, we also evaluated the effects of stirring times and thickness of the silver layer on the SERS detection activity of thiram. It was found that when the sharp tips of gold nanoparticles were not entirely covered by the Ag shell and still exposed to the outsides, the SERS performance was remarkably improved.

## Results and discussion

### Synthesis of gold nanostars (AuNSs)

#### Effect of seed-growth mixture stirred time on the formation of AuNSs

As the initial point for synthesizing the star-liked nanostructure, the seeds should be small nuclei and monodisperse, which could be achieved by instantaneously adding the reducing agent NaBH_4_ trisodium citrate under vigorous stirring. The obtained citrate-capped seeds colloidal solutions appear purple-red and present an absorbance peak at around 534 nm in the UV-vis spectrum (see Fig. S1†). This peak could be attributed to the surface plasmon resonance of spherical nanoparticles,^38^ which formed as aging for two hours to complete the decomposition of the remaining sodium borohydride in the solution.

In our method for synthesizing AuNSs, the seed colloids are kept under stirring for a fixed time in the growth solution before starting the particles growth to control the branches length and morphologies. The seed particles size gradually decreases once stirred in the growth solution due to participating in the con–proportionate reaction with Au^3+^ ions before forming star-liked nanoparticles.^39,40^ It was previously reported by Kawamura *et al.* that the very small citrate-stabilized seed particles could experience defective crystal growth to form star-liked morphology.^40^ However, this study focused on investigating the various stirred period of the seed-growth mixture (0, 0.17, 1, 2, 5, and after 70 minutes). Meanwhile, our preparation process for star-liked nanoparticles was investigated within the other periods (5, 10, 15, 20, and 25 minutes) of stirring the citrated seeds in growth solution before aging to achieve AuNSs with the longed well-formed branches.

1 2Au(s) + AuCl^−^_4_ → 3AuCl^−^_2_

**Fig. 2:**
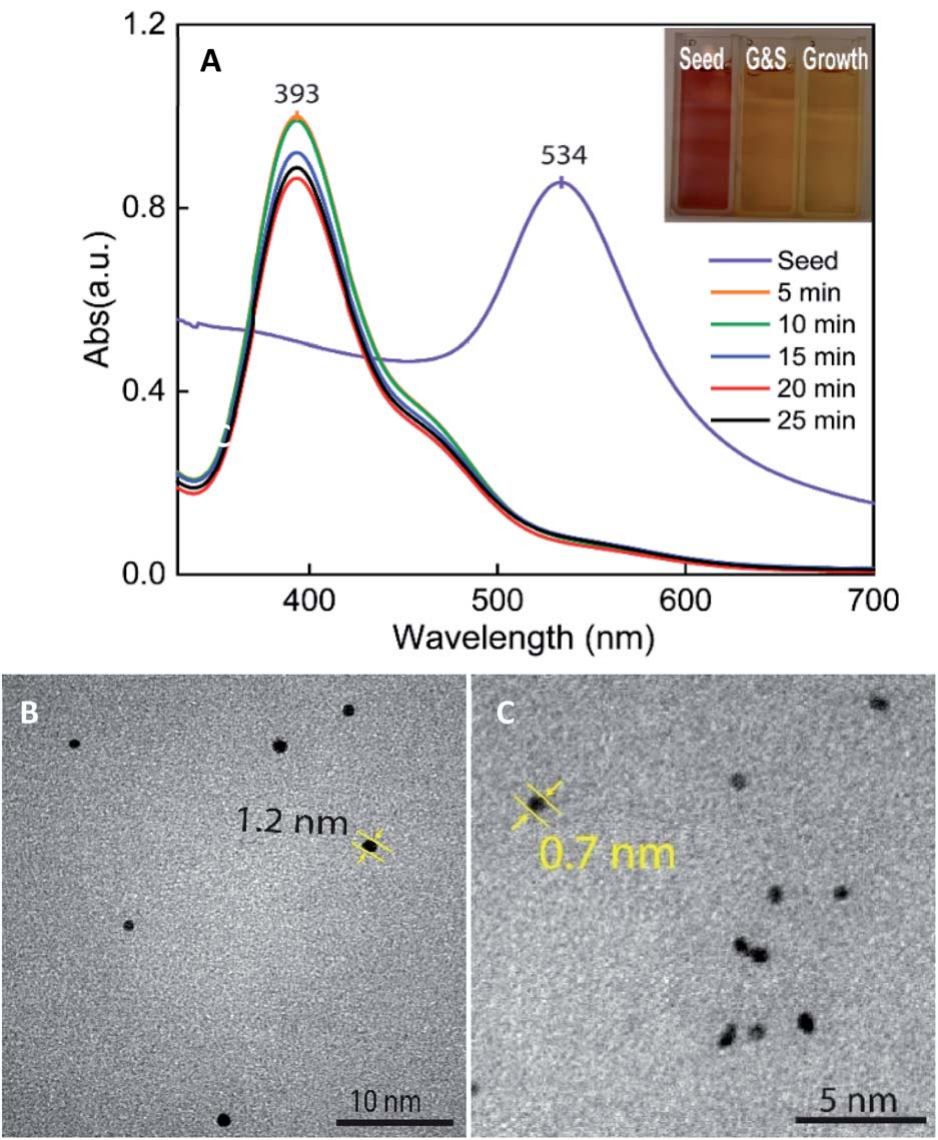
(A) UV-vis spectrum and corresponding digital photographs (right inset) of the as-prepared citrated-seed, and seed-growth colloidal solution stirred for different times from 5, 10, 15, 20, to 25 minutes. TEM image of citrate-stabilized seed particles stirred in the growth solution for (B) 10 minutes, and (C) 20 minutes.

The UV-vis spectrum of the seed-growth solution mixture exhibits the absorption peaks at 393 nm, which is assigned to the optical absorption of a complex of [AuBr_4_]¯-CTA.^41,42^ As shown in the spectral change exhibited in Fig. 2, there is a drastic blue-shift of the plasmonic peaks from the wavelength of 534 to 393 nm and an increase in the symmetry of absorbance band when mixing the preformed seed with the growth solution, indicating the oxidation of Au^o^ resulted in a decrease in particle size. It was previously postulated that particles sizes below 2 nm are known not to exhibit the surface plasmon resonance peak.^43^ During the con–proportionate reaction, the pre-formed seeds could be suffered the oxidation procedure, which lead to the formation of sub-nanometer particles. The transmission electron microscopy (TEM) results also support the con–proportionate reaction; it can be observed in Fig. 2B and C that the size of seed particles decreased to 1.2 nm and 0.8 nm after being stirred in growth solution for 20 and 25 minutes, respectively. In addition, the peak intensity is also decreased in the absorbance peak at 393 nm (Fig. 2A), which could be attributed to the decrease of Au(iii) ions (in the [AuBr_4_]¯-CTA complex). These results further confirm that the con–proportionate reaction occurs while stirring the citrated seeds in the growth solution. Besides, the addition of AgNO_3_ is necessary for assisting the anisotropic growth of Au branches on the multi-twinned citrate seeds. The Ag^+^ ions are preferentially absorbed on the (110) high energy facet of the gold nanoparticles and allow the Au atoms could deposit on their most energy favorable crystalline facet.^44^ It was proposed that Ag^+^ ions formed the chemical bonding with the Br^−^ ions of the surfactant, thus limiting the diffusion of Au^+^ ions to the (110) facets, which could promote the elongation of branches growth on the other crystal faces.^45,46^

The anisotropic optical properties of star-like gold nanoparticles mainly depend on the length and width of branches.^17,47^ The as-prepared AuNSs colloidal solutions with different stirred times of growth-seed solutions before adding ascorbic acid were characterized through UV-vis spectroscopy (see Fig. S2†). A plasmonic peak shift from 549 nm to 795 nm was observed as lengthened the seed growth stirred times for 0 to 20 minutes of stirred times, which attributed to the change from spherical to star-liked morphologies.^15,48,49^ Continuing to extend the stirred times for 20 and 25 minutes, broadband with low intensity is observed at a wavelength of 795 nm, and a long tail appears in the near-infrared region. The previous publications reported that the optical feature with the band located around 650 to 900 nm is known as distinct LSPR, which is strongly contributed by the nanostars branches and partly contributed from its core.^50,51^ The increase of branch length on gold nanostars thus leads to the red-shift in this LSPR band. The obtained colloidal solution of AuNSs is blue, and the intensity of the blue color also varies according to the stirring time (see Fig. S2,† right inset).

**Fig. 3:**
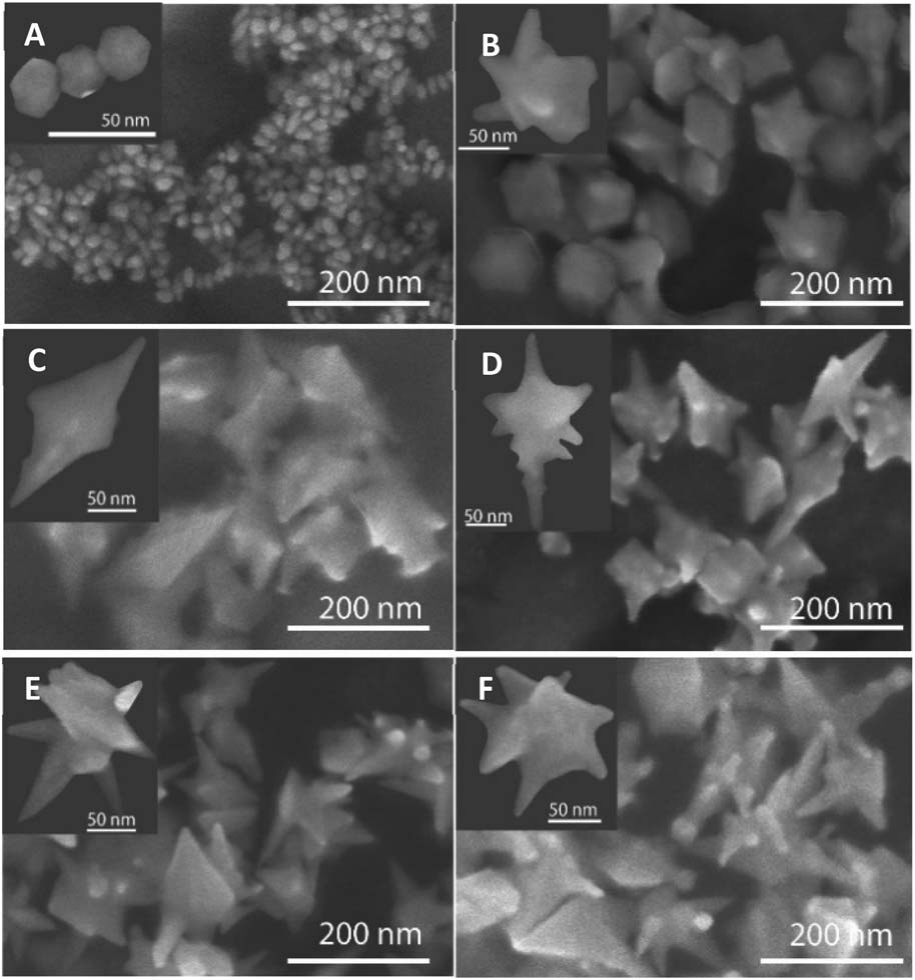
SEM images of AuNSs formed by the addition of ascorbic acid after stirring the seed-growth mixture for (A) 0 min, (B) 5 min, (C) 10 min, (D) 15 min, (E) 20 min, and (F) 25 min (scale bars for figures, 200 nm). The insets display the enlarged SEM images (scale bars for insets, 50 nm).

**Fig. 4:**
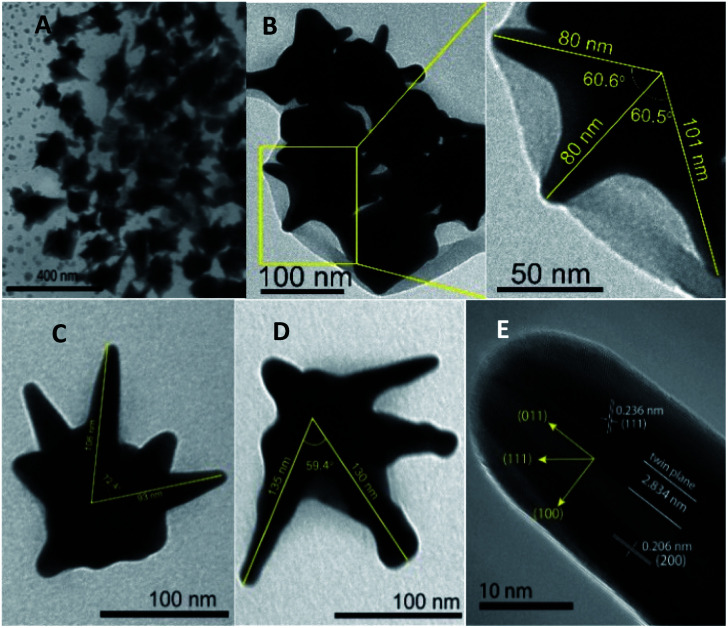
TEM images with the angle and core-to-tip measurement of AuNSs prepared by the addition of ascorbic acid after stirring the seed-growth mixture for (A) 15 minutes (scale bar 400 nm), (B) 15 minutes (a small field of view on several nanostars, scale bars 50–100 nm), (C) 20 minutes (scale bar 50 nm), and (D) 25 minutes (scale bar 100 nm). (E) High resolution TEM images with a small field of view on one-branch of the Au nanostars particle with a twinned planes. The scale bar represent 10 nm.

The microscopic observations support the UV-vis results, where the size and shape of particles are predicted to change as regulating the stirring time of seed-growth mixtures. For instance, each formed nanostructure is star-liked in shapes and possesses at least four to six branches, but branch lengths vary depending on different synthesis conditions. For the seed-growth stirring time of 5 minutes, the shape of each branch on the nanoparticles is relatively small and short (Fig. 3B) and begins extending from the middle section out to many directions. Fig. 3C and D displays the SEM images of the AuNSs obtained by adding ascorbic acid into the seed-growth mixture after stirring the citrate stabilized seed with the growth solution for 10 and 15 min. For 10 minutes of stirring times, some particles contain one more extended branch with a sharp tip than the others (Fig. 3C). The morphology of branches also reflects their origin from multiply twinned nanocrystal, with the existence of twin boundaries maneuvering along the long axes and the growth from the center section to form the two opposite sides (Fig. 3C left inset). The analysis of the HRTEM images also demonstrates the morphology of AuNSs. As shown in Fig. 4E, a twinned plane is visualized along the vertical of a branch on the star-like particle.

The formation of many AuNSs with relatively high surface roughness made up of many vertices and sharp edges is observed with a high ratio of particle population in the sample collected after 15 minutes of seed-growth mixing (Fig. 3D). Additionally, the sharp curvatures of the branches on particles are different depending on the change of seed-growth mixture stirring time. As observed in Fig. 3E, the nanoparticles prepared with the stirred time of seed-growth mixture of 20 min are well-developed star shape with an increase in the number of long branches that grows evenly in many directions, more equal in length and sharp vertices. The particle size was about 110 to 130 nm. Thus, the size and the number of the branches can be controlled by governing the seed-growth stirring times. Due to the longer stirring time of the seed-growth mixture, the size of the seeds becomes smaller, and the packing of the surfactant molecules on the nanoparticles is thus higher. Additionally, dense surfactant adsorption allows the seed particles to be closely capped in the micelle structure's soft template. That may cause the deposition rate of Au atoms into the CTA^+^ micelle cavity on the seed surface to be slower. This preferential adsorption of Au atoms on the remaining positions easily leads to the growth of AuNSs with long branches.^52^ A comparison SEM analysis of the sample prepared without stirring the pre-synthesized seeds in the growth solution was also conducted to confirm the role of the con–proportionate reaction time in controlling the shapes of nanoparticles. The microscopic image of the gold nanocrystal clearly shows that most nanoparticles formed are spherical or close to spherical (Fig. 3A).

There was an increase in the size of the AuNSs for the stirring times of 0, 10, 20, and 25 minutes, determined by the dynamic light scattering (DLS) method (see Fig. S3A–D†). Without stirring the seed-growth mixture, the nanoparticles have an average size of about 65 nm and broad size distribution. As stirring time progressed for 10, 20, and 25 minutes, the average size increased from 119 to 152 nm. The purity of the AuNSs was further investigated by energy-dispersive X-ray (EDS) spectroscopy, revealed that the AuNSs samples are mostly gold (82 weight%) and partially amount of Ag (18 weight%) (see the Fig. S3E†). TEM images of AuNSs at different magnifications obtained after stirring the seed-growth mixture for 10, 20, and 25 minutes are shown in Fig. 4. The angle between two adjoining tips and distance from the center of the particles to the top of the branches were measured using ImageJ software to define the size of gold nanostars. The average lengths from the core center of particles to the tip range from 80 to 130 nm when increasing the stirring time from 10, 20, and 25 minutes. At least four to six sharp tips are observed in the formed nanoparticles, and the angles between two adjacent tips range from 59.4 to 72.4° (Fig. 4C and D). The HRTEM technique was used for analyzing the insight structure of gold nanostars. The crystalline facets observed in the Fig. 4E indicate that the formed nanoparticles are highly crystalline. The *d*-spacing for the adjacent fringes of a tip on the nanocrystal can be measured at approximately 0.237 nm, which is assignable to (111) lattice space of gold metal. This result is similar to the previous report studied on the star-like gold nanocrystal with sizes ranging from 50 to 300 nm that gold nanoparticle tips are comprised of (111) outer faces.^53^

### Synthesis of gold nanostars coated with Ag (AuNS@Ag)

**Fig. 5:**
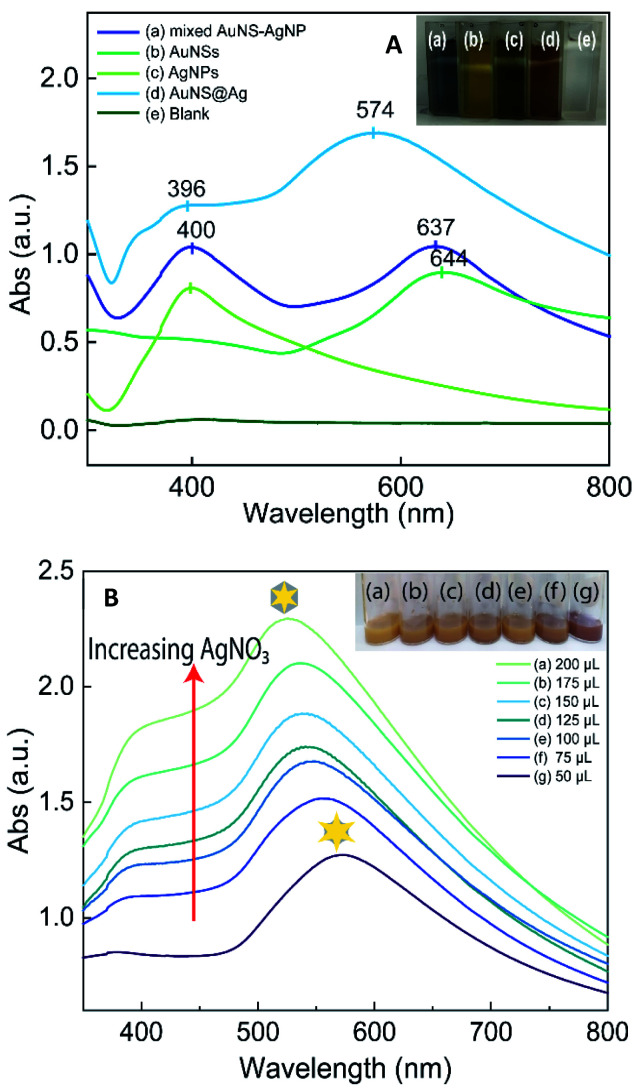
(A) UV-Vis spectra of the AuNS@Ag, AuNSs, AgNPs, physically mixed AuNSs–AgNPs, and the blank sample were prepared by adding 150 μL of 10 mM AgNO_3_ into the ascorbic acid solution without using the AuNSs. Corresponding digital photographs (right inset) of these colloidal solutions. (B) Compared UV-Vis spectrum of the prepared AuNS@Ag samples using different 10 mM AgNO_3_ volumes from 50 to 200 μL.

The coating of the silver layer over AuNSs was studied with a series of controlled experiments through varying the AgNO_3_ concentrations. In this section, the AuNSs samples collected with the addition of ascorbic acid after stirring the seed-growth mixture for 20 minutes were used in the Ag coating process. Besides, the UV-vis analysis of AuNSs, AuNS@Ag, and Ag nanoparticles colloidal samples prepared under the same condition were carried out to compare the plasmonic bands that appeared on each spectrum for investigating whether the reduction of AgNO_3_ occurs on the preformed AuNS surface rather than generating more nucleation sites. The spectrum of AuNSs coating with Ag colloidal solutions exhibits the broadband at 574 nm (Fig. 5A-curve blue), presuming the partial coverage of AuNS with silver. Compared to the absorbance spectra of AuNS samples (Fig. 5A-curve green), it can be found that silver deposited on the surface of AuNSs induced the blue shift of the SPR band from 644 to 574 nm. Simultaneously, the color of samples also obviously changed from its original AuNSs colloidal solutions dark-blue to yellow-orange (Fig. 5A-right inset). Besides, two separated plasmon bands that appeared around 400 and 637 nm were observed when physically mixing AgNPs with the AuNSs particles, attributed to the surface plasmon resonance of spherical silver nanoparticles and star-liked shape Au particles (curve dark blue). This result is also consistent with the blank spectrum of Ag nanoparticles that only one characteristic band appears around 400 nm (curve green). Fig. 5B provides the UV-vis absorption spectra of the AuNS@Ag colloidal solution taken at the different volumes of AgNO_3_ used in the coating process. The absorbance intensity increases progressively when more Ag^+^ is added to the AuNSs and ascorbic acid mixture. For 50 μL of AgNO_3_, the absorbance feature consists of the plasmonic band at 573 nm and the shoulder band at 380 nm. Interestingly, the increased volume of AgNO_3_ in the reaction system gives the slight blue shift in the absorption band at 573 to 526 nm and rise to the redshift in the maximum wavelength at 380 to 405 nm. The band approximately at 550–650 nm is known as the transverse absorption mode of the branch on star-liked shape.^53^

The nanoparticle morphologies evolution during the coating process were further characterized using SEM (see Fig. S4†). It is observed that the morphology of the preformed AuNSs was relatively changed; the sharp edges on star-liked nanostructure have become more rounded. The majority of AuNS@Ag nanoparticles are not identical to star-liked in the shape of the gold core before coating with silver. The branches become rounder with the blunt tips, but small protrusions on the vertices can be visible (see Fig. S4†). From the above UV-vis and SEM analysis, it can be found that an appropriate volume of AgNO_3_ is essential for AuNS@Ag particles to preserve their star-liked shapes. However, a high volume adding of Ag^+^ can form nanoparticles close to spherical and polyhedral shapes. Overall, at the 100 μL volume of AgNO_3_ with ascorbic acid and preformed AuNSs, the AuNS@Ag can be obtained after 20 minutes of the coating process.

#### X-ray diffraction of AuNSs and AuNS@Ag

To further examine the crystalline structure of AuNSs and AuNS@Ag, the X-ray diffraction of the precipitation fraction of nanoparticles after centrifugation was taken (see Fig. S5†). The diffraction peaks depicted that the synthesized AuNSs are highly crystalline gold; no other impurities peaks are found in the XRD pattern. The *d*-spacing and the lattice constant value (*a* = 4.077 Å) can be determined from the XRD data based on Bragg's equation^54^ and the cubic crystal system^55^ (see Table S1†). In comparing AuNS and AuNS@Ag X-ray diffraction patterns, there is no significant difference in the position of the characteristic peaks. Nevertheless, the AuNS diffractogram shows a higher peak in 2*θ* value of 38.2° compared with the AuNS@Ag pattern, which could be due to the deposited Ag nanostructure on the Au core (see Fig. S5†).

#### TEM and EDS of AuNS@Ag characterization

**Fig. 6:**
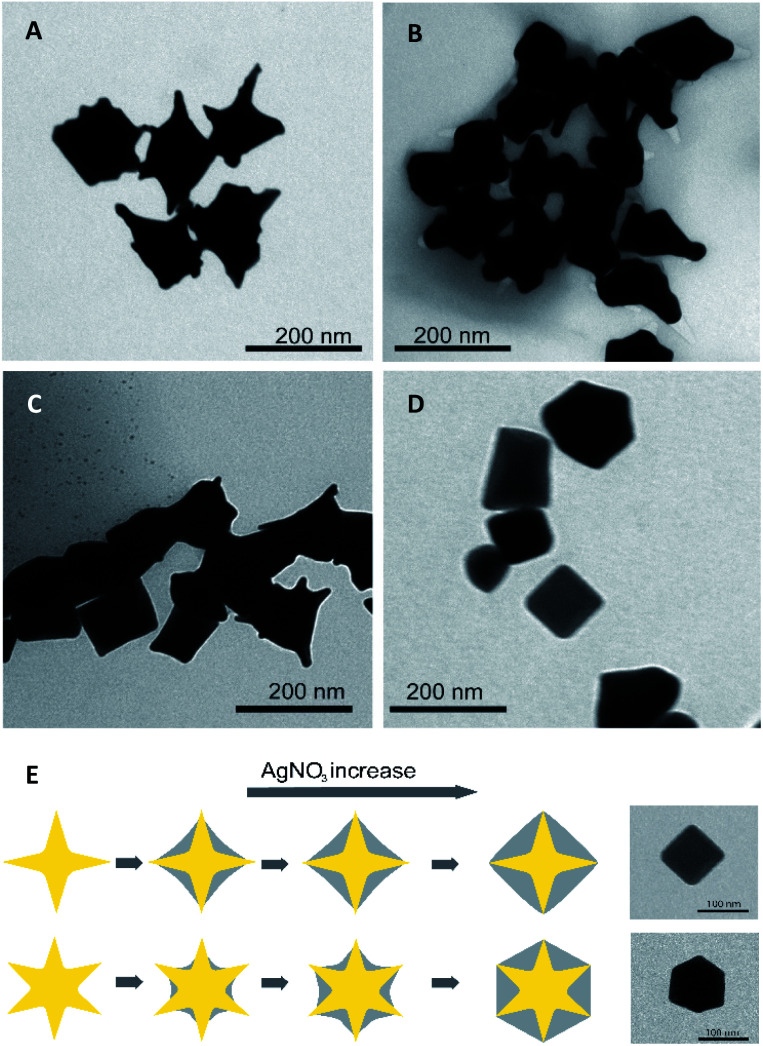
TEM images of Ag-coated AuNSs prepared with (A) 50, (B) 100, (C) 150, and (D) 200 μL AgNO_3_ 10 mM (scale bars for all, 200 nm). (E) Scheme for the growth of Ag layer on AuNS nanoparticles (scale bars for insets, 100 nm).

The morphology and size of the formed AuNS@Ag nanoparticles are analyzed using the TEM technique. The TEM images of Ag coated AuNSs using the different volumes of AgNO_3_ solution 10.0 mM are shown in Fig. 6. It can be seen that Ag is merely deposited on the surface of AuNSs and the sharp tips are remained exposed to the outside at the low addition volume of 50.0 μL AgNO_3_ solution. The formed angles between two adjoining tips are gradually wider and tend to evolve into a straight line with the addition of more AgNO_3_ volume (Fig. 6B). As increased to 200 μL volume of AgNO_3_ solution, the gold nano-stars are more wholly encapsulated in the Ag shelled (Fig. 6D). The particles seem to have morphologies like polyhedral shapes when observed in the TEM image (Fig. 6D). The results of the TEM characterization coincide with the change of plasmonic properties on the UV-vis spectrum for the different volume adding of AgNO_3_.

HRTEM technique was further used to determine the morphology of AuNS@Ag. The high contrast between the center and periphery reveals the coating of the Ag layer on the AuNSs structure. The Ag layer partly covered the Au star-like nanoparticle, but the end of the AuNS sharp tip remains exposed (see Fig. S6B†). The high contrast results also suggest that the nanoparticles cannot be composed of pure Au or Ag. In this case, the contrast between the Au core and Ag layer could not appear.^56,57^ The *d*-spacing of the adjacent fringes near the nano-star core was 0.235 nm, which could be attributed to Ag (111) lattice space. However, the *d*-spacing values clearly observed on the end of the AuNS tip are 0.238 and 0.14 nm, which are assigned to the (111) and (220) planes of Au. This observation result strongly suggests that the area near the particle core was covered by Ag (111) crystal facet, so we cannot observe the other planes of Au metal. Furthermore, on the vertex of the tips on AuNS@Ag, which is not embedded in the Ag layer, the (002) plane is still seen in the local enlarged HRTEM image. Selected-area electron diffraction (SAED) patterns analysed from one particle showed the crystallinity of AuNS@Ag particles with intensive diffraction spots (see Fig. S6E†). The ring diffraction patterns indicated that the AuNS@Ag nanoparticles are polycrystalline materials.^58^ The SAED observations are in accordance with XRD data. To better observe AuNS@Ag local composition, EDS elemental mapping and spectrum characterization of AuNS@Ag particles was conducted (see Fig. S7†). At the addition of AgNO_3_ 10 mM volume of 150 μL, the Ag was preferentially deposited onto the AuNSs surface, and the gold nanoparticles retained their morphology with the sharp tips.

More information about the surface composition of AuNS@Ag nanoparticles can be further provided by X-ray photoelectron spectral (XPS) analysis. The XPS results showed that Au, Ag, Cl, O, and C elements exist in the AuNS@Ag nanostructure. Both Au 4f (84.16 eV) and Ag 3d (367.36 eV) peaks are observed on the AuNS@Ag nanoparticles XPS analysis, which is subtly different from the literature data^59^ (see Fig. S8†). The atom percentage results suggested more enrichment of Ag on the surface of the nanoparticles (see Table S2†).

### SERS characterization

The star-like nanostructures provide a large cross-section for better SERS performance than spherical morphology due to the hybridization of plasmons localized at the core and the tips of nanoparticles.^60^ Moreover, it is beneficial when combining the stability of gold nano-stars with the stronger plasmon resonances of silver shells in enhancing the SERS effect. The Raman spectra of pure thiram; thiram at the concentration of 0.1 ppm investigated with AuNS, AuNS@Ag, and blank sample prepared with AuNS@Ag substrate are taken for studying the SERS performance. The analysis results clearly show that the AuNS@Ag structure enhanced the SERS performance. (See Fig. S9†). AuNS@Ag substrate yields better results with the higher intensity of Raman peaks located at 472, 556, 1380, 1509, and 2920 cm^−1^ than AuNSs for detecting thiram. In detail, the intensity of peak at 556 cm^−1^ is decreased for AuNS@Ag substrate compared to the Raman spectrum of thiram powder; this change might be explained due to the cleavage of the S–S bond thiram. The cleavage of the S–S bond could cause the formation of the resonated radical structure, which leads to the appearance of the new Raman peak at 1509 cm^−1^ assigned to the C–N vibration asymmetric stretching vibration^61^ and increases the intensity of the peak assigned for the C=S vibration located at the Raman shift of 472 cm^−1^.^4,61,62^ Noteworthy, the Ag surface was found to form a chemical interaction with thiram, resulting in the S–S bond cleavage through methyl group and S atom.^61^

**Fig. 7:**
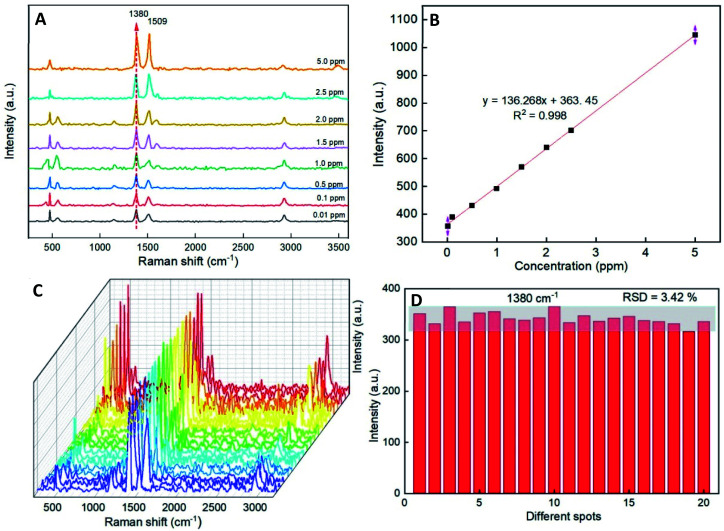
(A) Typical SERS spectra for different concentration of thiram tested with AuNS@Ag. (B) Calibration curve of thiram. All experiments were conducted at room temperature. (C) A serial SERS spectra of thiram (0.5 ppm) molecules from 20 different spots on the AuNS@Ag, and (D) the Raman vibrational intensities of thiram (0.1 ppm) at 1380 cm^−1^.

In order to evaluate the Raman signal enhancement of the AuNS@Ag substrate, a series of experiments with various concentrations (from 0.01 to 5.0 ppm) of thiram was conducted. Fig. 7A shows the SERS spectra of various concentrations of thiram. Most of the characteristic peaks at 472, 556, 1380, and 1509 cm^−1^ are obviously observed at different concentrations of thiram ranging from 0.01 to 5.0 ppm. In particular, the high-intensity peak at 1380 cm^−1^ assigned to C–N stretching vibration was selected as the prominent characteristic peak, and its intensity increased concomitantly with the rise of thiram concentration. Fig. 7B exhibits the calibration of different thiram concentrations, which showed a high *R*^2^ of 0.998 for the thiram in the concentration range from 0.01 to 5.0 ppm. The limit of detection (LOD) for thiram is 0.22 ppm, and the limit of quantification (LOQ) is 0.73 ppm. The results further demonstrate that the as-prepared AuNS@Ag substrate has a good improvement effect on SERS performance.

The reproducibility of signals from SERS spectra is an essential routine analytical tool for detecting organic residue. To verify whether the substrates can provide reproducible Raman signals at a low concentration of thiram molecules, SERS spectra of thiram molecules with the concentration of 0.1 ppm from 20 randomly spots on the substrate have been investigated. The results confirmed that the AuNS@Ag substrates have good reproducibility and the RSD values from 20 spots at 1380 cm^−1^ are 3.42% (Fig. 7C and D).

### The mechanism of SERS enhancement

The good SERS enhancements are obtained from single isolated nanoparticles or the space of high field gradients between two particles.^2^ In the case of AuNS@Ag nano substrate, thiram can be adsorbed onto the silver layer surface or the AuNSs tips by forming a soft bonding of the S atom in the S–C groups.^34^ As adsorbed onto the silver layer, Raman intensity amplification of the analyte was closely related to the thickness of silver layer. It was proposed that the surface would be oxidized to generate an Ag^+^ ion in the presence of oxygen. A coordinate bond can be formed with donor groups and generated molecular Ag^+^ complexes on the surface.^2^ The effect of silver layer thickness on the SERS detection of thiram was also investigated (see Fig. S10†). The results showed that the Raman characteristic peaks for thiram are remarkably enhanced when using AuNS@Ag nano substrate of suitable Ag layer thickness. Using particles with a too thick coating will reduce the Raman peak intensity, which partly elucidates the SERS enhancement mechanism of AuNS@Ag nano substrate. Besides, thiram molecules can be interspersed in the voids created by the specific morphology of AuNSs, which were previously known as hot spots. The roughness of star-like gold nanoparticles also affects the SERS performance of thiram detection. That was previously known to relate to the plasmon energy, which differs between the smooth and rough surfaces.^2,63^ A serial SERS measurement on the samples obtained by changing the stirred time of the seed-growth mixture was conducted to clarify the particle's surface roughness effect on Raman peaks amplification (see Fig. S10†). As lengthen the stirring time, the formed particles will have more sharp tips with the length increase, and the intensity peaks arise concomitantly.

## Conclusions

The sharpness and length of gold nano-stars branches have been successfully controlled through a convenient seed-growth method by governing the stirred time of the seed-growth mixture. The branches of AuNSs grow longer when stirred in the seed-growth mixture for about 20 to 25 minutes before adding ascorbic acid. Besides, the seed particle sizes and dispersity are valuable conditions for the controlled synthetic of tips on AuNSs. The amount of citrate and aging times served effectively for forming 4 nm diameter colloids seeds. The interconversion of AuNSs morphologies was studied by SEM, TEM, and HR-TEM. The EDS spectrum and SAED exhibited a good association with the XRD pattern about the crystalline structure of the formed gold nanoparticles.

Our study demonstrated that the AuNSs could be effectively coated in the silver shells with an appropriate silver concentration and varying the thickness of the Ag shell. The AuNS@Ag are prepared *via* the initial formation of Au star-liked templates with multi-spike morphologies, and these plasmonic structures could possess many hot spots to enhance the Raman signals of thiram molecules significantly. SERS was successfully applied to detect thiram with the range from 0.01 to 5.0 ppm. These finding reported here could extend on quantification of thiram using SERS nano-substrates in the food industry.

## Experimental section

### Materials

Chloroauric acid (HAuCl_4_·4H_2_O) was purchased from Sinopharm Chemical Reagent Co., Ltd. (Beijing, China). Silver nitrate (AgNO_3_, 99.0%), L (+)-ascorbic acid (C_6_H_8_O_6_, 99.0%), sodium borohydride (NaBH_4_, 96.0%), trisodium citrate (TSC, 99.0%), and DI water (>18 MW, Millipore, conductivity <4.3 μS cm^−1^) were obtained from Sigma-Aldrich (Darmstadt, Germany). Hydrogen peroxide (H_2_O_2_, 30.0%) and sodium hydroxide (NaOH, 96.0%) were obtained from Xilong Scientific Chemical Reagent Co., Ltd. (Shantou, China). Cetyltrimethylammonium bromide (CTAB, 99.0%) was purchased from HIMEDIA (Mumbai, India). Thiram (C_6_H_12_N_2_S_4_, 97.0%) was purchased from AK Scientific, Inc. (Union City, USA). All reagents were used without further purification, and the aqueous solution experiments were prepared using DI water. All glassware and magnetic stirring bars were cleaned with aqua regia (HCl/HNO_3_, volume ratio 3 : 1) and thoroughly rinsed with Millipore water (conductivity <4.3 μS cm^−1^) several times prior to use in all experiments.

### Preparation of gold nanostars (AuNSs) and gold nanostars coated with Ag (AuNS@Ag) by the seed-mediated modification method

#### Preparation of seed nanoparticles

The Au seeds were synthesized according to the seed growth approach derived from the citrate-reduction method by reducing HAuCl_4_ with NaBH_4_ at room temperature. Briefly, A 9.90 mL aliquot of 0.25 mM HAuCl_4_ was added into a 10 μL of freshly prepared 0.25 M TSC aqueous solution under magnetically stirred at the rate of 400 rpm. The above mixture was added to 30 μL of ice-cold NaBH_4_ aqueous solution of 0.1 M, followed by gentle stirring for 1 minute. The colloidal seed solution was left undisturbed for 2 hours to decompose the borohydride and was best used within 2–6 hours after preparation. The color of the solution gradually turned from pale yellow to bluish purple and was further used to prepare gold nanostars.

#### Controlled synthesis of gold nanostars (AuNSs)

The growth process of Au nanostars involves the reduction of chloroauric acid (HAuCl_4_) with ascorbic acid at ambient temperature in the presence of as-prepared seed colloid, the cationic surfactant CTAB, and additional additives. A typical procedure is as follows: 3.6445 g CTAB was added into 70 mL DI water followed by vigorous stirring within 5 min for complete dissolution. Afterward, 1.0 mL of 25.0 mM HAuCl_4_ and 50 μL of 0.1 M AgNO_3_ were mixed in the as-prepared CTAB aqueous solution; and the volume was adjusted to 100 mL with DI water. The growth solution contained 0.25 mM HAuCl_4_, 0.10 M CTAB, and 50 μM AgNO_3_. Separately, 10 mL of growth solution was further mixed with 0.8 mL of seed solution and continued stirring for 15 min at a rate of 400 rpm. Then, A 50 μL aliquot of freshly prepared 0.1 M ascorbic acid was added dropwise into the above mixture, continuously stirring for 30 seconds. The pale yellow color of the seed-growth mixture changed to almost colorless after adding ascorbic acid, indicating the reduction of Au^3+^ to Au^+^ species, and turned to dark blue within 15 min. The colloidal solution was aged for 2 hours at room temperature to complete the formation of the star-liked gold nanoparticles. The obtained colloids do not show precipitation and are remarkably stable in three months.

The resulting colloidal solutions were centrifuged at 8500 rpm for 10 min using the Microfuge 16 Centrifuge (Beckman Coulter, Switzerland), followed by replacing the supernatant fluid with Mili-Q water to remove excess cationic surfactant and L-ascorbic acid. This substitution procedure was replicated three times to obtain the AuNSs further used in the Ag coating process.

#### Coating gold nanostars with Ag shells (AuNS@Ag)

After centrifuging the as-prepared AuNSs colloids, the precipitate fraction was rinsed with 3.0 mL of DI water three times and then re-dispersed by ultrasound for 10 min. Afterward, a 270 μL of freshly prepared 10.0 mM ascorbic acid was added to the above mixture, followed by shaking for 90 seconds. Subsequently, a 150 μL volume of 10 mM AgNO_3_ was added dropwise into the colloidal solution at a rate of one drop per 15 seconds. The obtained colloids were continuously shaken for 20 minutes to complete the coating process. The silver shells grew on the surface of AuNSs *via* the reduction of silver nitrate by acid ascorbic, and the shell thicknesses depended on the concentration of AgNO_3_. The color of the solution changes slowly from dark blue to yellow-orange, indicating the formation of a silver shell on the AuNSs. The as-prepared colloids were then centrifuged at the rate of 6000 rpm and rinsed double times with DI water to remove the reagents residue and enhance the long-term stability of AuNSs.

### Characterization of AuNSs and AuNS@Ag

UV-vis absorption spectra of the colloidal samples were collected on the UV-vis-NIR-V670 spectrometer (Jasco, Japan) using quartz cuvettes (1 cm path length) under the testing range of 300–800 nm, the scanning rate of 200 nm per minute. SEM measurements were conducted on a JEOL JSM-7600F (USA) equipped with an energy-dispersive X-ray spectroscopy (EDS) receiver: Oxford Instruments 50 mm^2^ X-max (UK) and a cathode fluorescence detector (CL) Gatan Mono CL4 (UK). To separate AuNSs from the colloids for SEM determination, the freshly prepared colloidal solutions were concentrated by centrifugation of nanoparticles at 6000 rpm for 10 min. The supernatant fluid was subsequently discarded, and the precipitation was rinsed with 200 μL of Mili-Q water. The rinse process was replicated three times to remove the residual surfactant. The precipitate was re-dispersed by adding 100 μL of Mili-Q water to each Eppendorf. Particle sizes were measured with ImageJ processing software, and the size of 100 particles was statistically determined to obtain the size distribution. SEM images were calibrated based on their corresponding scale bar, and at least ten images were taken per sample. The shape and size of nanoparticles were further analyzed by transmission electron microscopy (TEM) using a JEM-2100 (JEOL Ltd, Japan) at an acceleration voltage of 200 kV. Before TEM analysis, the samples were prepared by pipetting 20 μL of the ultrasonic disperse sample onto the carbon-coated copper mesh (300-mesh, Ted Pella, Inc, Redding, CA, USA) then dried at ambient temperature. The particle morphology obtained on TEM images was determined by estimating the core-to-tip, core diameter, and angle of the two adjacent tips (measured with ImageJ software) based on their respective scale bars. The average size of AuNSs and AuNS@Ag particles was also determined by the dynamic light scattering (DLS) method using a Horiba SZ-100 (Horiba Ltd., Japan). Selected area electron diffraction (SAED) was performed in a Jeol-2100 (JEOL Ltd., Japan). High-resolution electron microscopy (HRTEM) was conducted on a JEOL ARM 200F (Jeol Ltd, Japan), generated at an acceleration voltage of 80–200 V. Energy-dispersive X-ray spectroscopy (EDS) of obtained AuNSs and AuNS@Ag was determined using a JEOL JSM 7600F (Jeol Ltd., Japan) at 30 kV for analyzing local composition. X-ray diffraction (XRD) pattern was collected on a D8 Advance-Bruker (Germany) to study the crystalline structure of gold nanoparticles. The as-prepared nanoparticles were concentrated by centrifugation of the colloidal solutions at 6000 rpm for 10 min and rinsed with DI water three times; the obtained precipitate was then vacuum dried and placed on a glass sheet for XRD analysis at a scanning rate of 0.05° per min in the 2*θ* range from 20° to 80°. The chemical compositions of the samples were determined using an X-ray photoelectron spectroscopy (XPS; Thermo Scientific, Waltham, MA, USA) instrument with a monochromatic Al Kα X-ray source at the photon energy of 1486.7 eV.

#### Preparation of SERS sample

Thiram was used as the probe molecule to determine the SERS enhancement properties and reproducibility of the AuNS@Ag nano-substrate. The stock solution of thiram (100 ppm) was prepared in ethanol before being diluted to various concentrations of 0.01, 0.1, 0.5, 1.0, 1.5, 2.0, 2.5, and 5.0 ppm of thiram. The pH of each solution was controlled as 4.0 using HCl 0.1 M. The pre-synthesized AuNS@Ag was washed by centrifuging at 6000 rpm for 10 min and then re-dispersed in 1.0 mL ethanol. Firstly, microscope glass slides were immersed in aqua regia (HNO_3_/HCl, 3 : 1 volume ratio) for 2 hours and thoroughly washed with DI water before being used. A 10.0 mL volume of AuNS@Ag colloidal solution was deposited onto a clean glass slide and then air-dried to form a thin film substrate and evaporate the residual ethanol. Subsequently, 20 μL of thiram solution with different concentrations was dropped onto this thin-film substrate and naturally dried at ambient temperature for 10 minutes. The as-prepared glass slides were then used for the SERS analysis. The SERS sample using solely AuNS as nano-substrates was also prepared in the same condition for the comparison experiment.

#### SERS spectral collection

Raman measurements of all the samples were performed using the XploRa PLUS confocal Raman Microscope (Horiba France SAS, Longjumeau, France) operated at 532 nm of an excitation source and 50 mW laser power. All spectra were collected with ×50 objective from 400 to 3500 cm^−1^ of wavelength. The laser power was 100%, with the exposure time set up at 15 seconds and accumulation times of 2. The 1 mm inner diameter capillary tube was used to graft samples. The Raman spectra were analyzed with LabSpec 6 software suite (Horiba France SAS). In some cases, the original spectra were modified by preprocessing steps, including de-spiking, denoising, and baseline correction.

#### Data analysis

The thiram limit of detection is calculated by using equation:^64^2
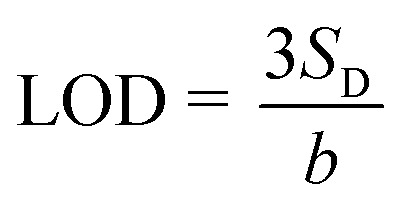


*S*_D_ is denoted as the standard deviation of the SERS intensity of the Raman bands of 1380 cm^−1^, and *b* is the slope of the calibration curves. Besides, the limit of quantitation (LOQ) was determined with the equation:3
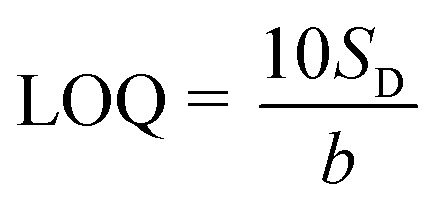


## Author contributions

Conceptualization, A. T. N. Q., T. A. N., S. V. V., I. P., and K. Q. V.; methodology and analysis, T. N. H. L, I. P. Investigation, T. A. N., N-A. T. Q., S. V. V., T. N. H. L., I. P., and K. Q. V. writing-original draft preparation A. T. N. Q., T. A. N., K. Q. V.; writing-review and editing, I. P., and K. Q. V.; supervision, K. Q. V. All authors reviewed the manuscript.

## Conflicts of interest

There are no conflicts to declare.

## Supplementary Material

RA-012-D2RA03396H-s001
