# Supplementary material for: Facile tuning of tip sharpness on gold nanostars by the controlled seed-growth method and coating with a silver shell for detection of thiram using surface enhanced Raman spectroscopy (SERS)
Source: RSC Adv. 2022 Aug 15;12(35):22815–25. doi: 10.1039/d2ra03396h (PMC9376760; doi:10.1039/d2ra03396h)
Supplement: RA-012-D2RA03396H-s001 [file RA-012-D2RA03396H-s001.pdf]

Electronic Supplementary Material (ESI) for RSC Advances.
This journal is © The Royal Society of Chemistry 2022

RSC Advances

SUPPLEMENTARY INFORMATION

Received 00th January 20xx,
Accepted 00th January 20xx

DOI: 10.1039/x0xx00000x

FACILE TUNING OF THE TIPS SHARPNESS ON GOLD NANOSTARS BY CONTROLLED SEED-GROWTH METHOD AND COATING WITH SILVER SHELL FOR DETECTION OF THIRAM USING SURFACE ENHANCED RAMAN SPECTROSCOPY (SERS)

Anh Thi Ngoc Quang[a], Thu Anh Nguyen[b], Sy Van Vu[b], Tien N. H. Lo [c], In Park*[c,d], Khuong Quoc Vo*[b].

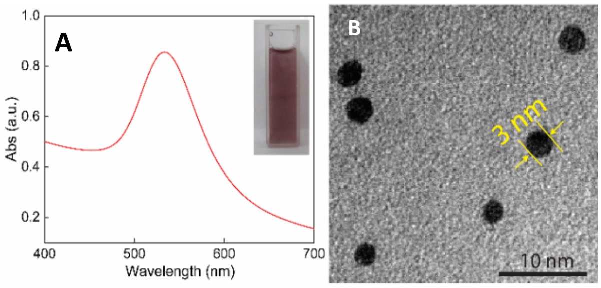

Figure S1. (A) UV-Vis spectra, corresponding digital photographs (right inset), and (B) TEM image with corresponding histogram (right inset) of the citrate stabilized seed nanoparticles.

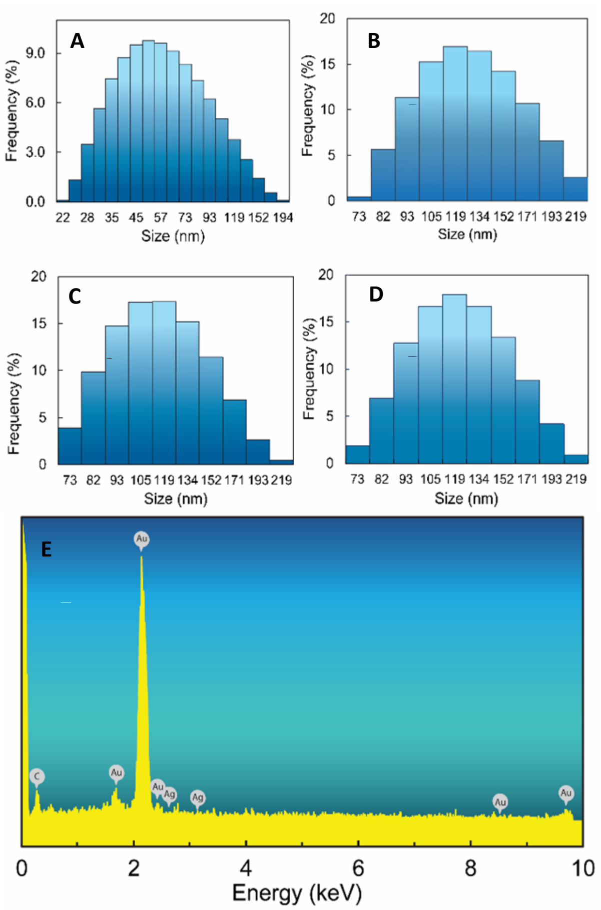

Figure S3. Size distribution histograms measured by dynamic light scattering (DLS) of the AuNSs formed after (A) 0 min, (B) 10 min, (C) 20 min, and (D) 25 min of stirring the seed-growth mixture. (E) Energy dispersive X-Ray (EDS) elemental spectrum of AuNSs.

The micrographs of AuNS@Ag samples collected after introducing various volumes of 10 mM $AgNO_3$ to the colloidal solutions containing AuNS premixed with ascorbic acid are shown in Figure S4. By coating the AuNSs with 100 µL of 10 mM $AgNO_3$, the nanoparticles increased in size by mainly 100-150 nm (See Figure S4-A), but the tips can still be distinguished. Some particles are discernible as the bipyramid morphology. However, when increased to 200 µL of 10 mM $AgNO_3$, the morphology of some particles observed on the SEM images has a significant conversion (Figure S4-C). The nanoparticles size slightly increased to 160 nm after 20 minutes of coating.

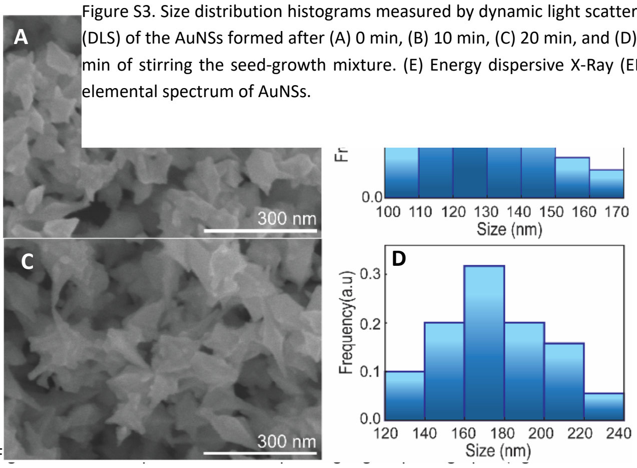

Figure S4. SEM images of AuNS@Ag nanoparticles formed by adding (A) 100 and (C) 200 µL (scale bar for all, 300 nm) of 10.0 mM $AgNO_3$ into the colloidal solution containing AuNSs and ascorbic acid. (B), (D) corresponding size distribution histograms of the AuNS@Ag samples prepared with 100 and 20 µL $AgNO_3$ 10.0 mM, respectively.

[a.] *Institute of Applied Technology, Thu Dau Mot University, 6 Tran Van On Street, Phu Hoa Ward, Thu Dau Mot City, Binh Duong Province, Vietnam.*
[b.] *Faculty of Chemistry, Ho Chi Minh City University of Science, Vietnam National University - Ho Chi Minh City, Ho Chi Minh City, 227 Nguyen Van Cu Street, Ward 4, District 5, Ho Chi Minh City 70000, Vietnam.*
[c.] *Research Institute of Clean Manufacturing System, Korea Institute of Industrial Technology (KITECH), 89 Yangdaegiro-gil, Ipjang-myeon, Cheonan 31056, South Korea.*
[d.] *KITECH school, University of Science and Technology (UST), 176 Gajeong-dong, Yuseong-gu, Daejeon 34113, South Korea*

** Corresponding author*
Email addresses: vqkhuong@hcmus.edu.vn (Khuong Quoc Vo),
inpark@kitech.re.kr (In Park)

XRD characterization

The XRD pattern of AuNSs is determined with four diffraction peaks in 2ϴ values of 38.2°, 44.4°, 64.7°, and 77.6°, which are assigned to the (111), (200), (220), and (311) planes of face-centered cubic gold with the Joint Committee on Powder Diffraction Standard (JCPDS) card number 00-004-0784, respectively. The prominent diffraction peak ascribed to the (111) planes centered at 38.2°, compared with (200), (220), and (311), showing that the (111) plane is likely the predominant orientation of the gold nanocrystals.

The Bragg equation:

$$n\lambda = 2d\sin\Theta$$

Where λ = 1.5406 Å has denoted the wavelength of the radiation used, ϴ is the Bragg diffraction angle (in Radian), n=1 is assigned to the order of diffraction, and d-spacing is interplanar spacing (in Å). The equation is applied to the major peaks observed on the pattern.

Table S1. The experimental XRD data and calculated d-spacing of the AuNSs sample.

(200)	44.403	22.202	2.038
(220)	64.734	32.367	1.439
(311)	77.640	38.820	1.229

These typical bright diffraction spots corresponding to the (111),

(200), (220), (311),

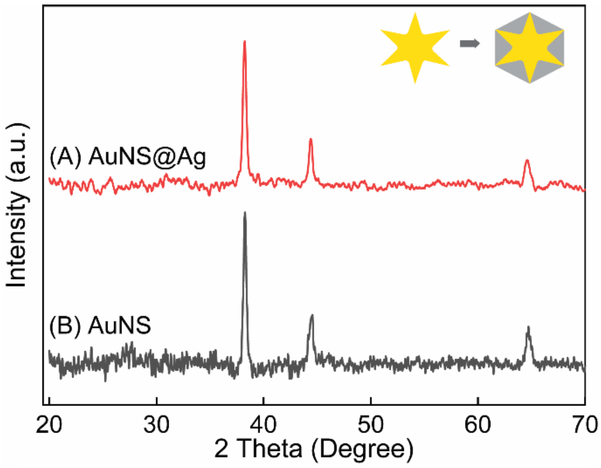

Figure S5. XRD pattern of (A) AuNSs synthesized with the seed-growth mixture stirred time of 20 minute, and (B) AuNS@Ag prepared with 200 µL $AgNO_3$ 10 mM.

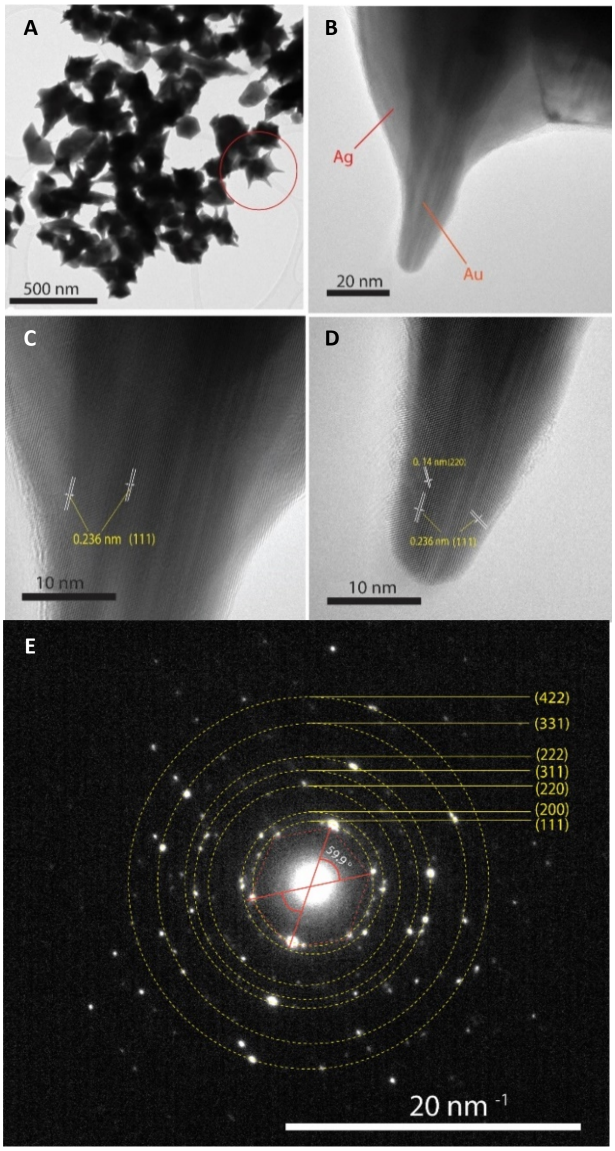

Figure S6. (A) TEM image, (B) high-resolution TEM image taken from the Ag shell area of AuNS@Ag nanoparticles (150 µL $AgNO_3$ 10 mM), HRTEM images with (C) d-spacing of Ag fcc crystalline-structure, (D) d-spacing of AuNSs, and (E) corresponding SAED pattern of AuNS@Ag prepared with the seed-growth stirring time of 20 minutes.

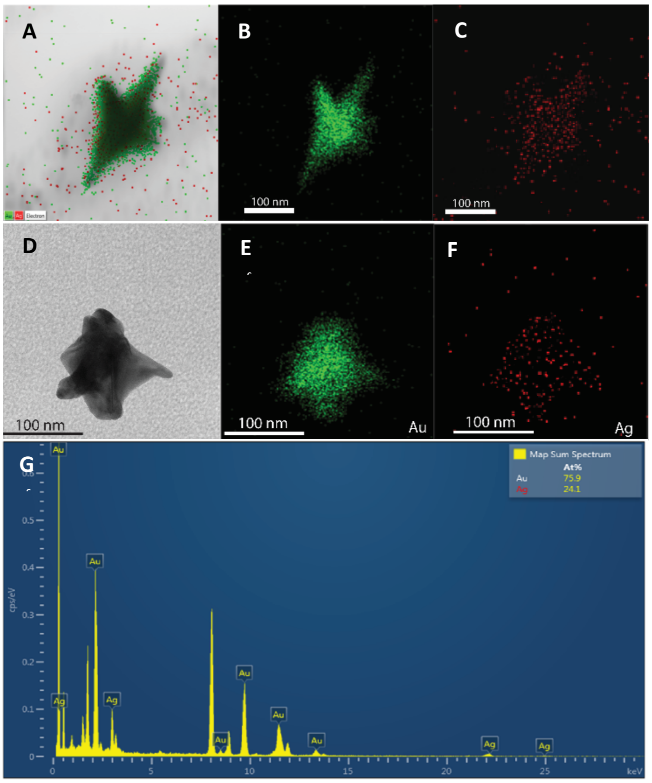

Figure S7. (A) The superimposed image TEM-EDS elemental mapping of Au and Ag on the AuNS@Ag particle (150 µL $AgNO_3$ 10 mM). The separated EDS mapping of (B) Au and (C) Ag elements, respectively. (D) TEM image of AuNS@Ag (200 µL $AgNO_3$ 10 mM). The separated EDS mapping of (E) Au and (F) Ag elements, respectively. (G) the EDS spectroscopy Au and Ag.

This journal is © The Royal Society of Chemistry 20xx

(222), (331), and (422) are also observed. Moreover, by measuring the radius of each ring with the scale on the picture, we can determine the interplanar spacing (See Figure S6-E).

EDS characterization

Figure S7 B-C and E-F displayed the element distribution of AuNS@Ag coated with 150 and 200 μL AgNO3 10.0 mM, respectively, with the green color indicating Au and the red color showing the distribution of Ag elements. The high-intensity signals around 2, 8, 10, and 11 KeV in the EDS spectrum confirm the presence of Au nanoparticles. This result is also similar to previous report (Figure S7 G)[1].

XPS characterization

The percentages of individual elements were determined based on the peak areas of the relative bands and their sensitivity factors. The high-resolution XPS spectrum of Au 4f is shown in Figure S8-B. At 83.79 and 87.48 eV, two peaks were observed assigned to Au $4f_{7/2}$ and Au $4f_{5/2}$, respectively. In the high-resolution XPS spectrum of Ag 3d, the two peaks obtained at 367.34 and 373.34 eV belong to Ag $3d_{5/2}$ and Ag $3d_{3/2}$[2].

Table S2. XPS peak position (BE/eV) for AuNS@Ag

	Binding energy	Literature data [2]	Atom %
Au 4f	84.16		41.07
Ag 3d	367.36		47.02
Au $4f_{7/2}$	83.79	84.0	0.78
Au $4f_{5/2}$	87.48	88.0	1.45
Ag $3d_{5/2}$	367.34	368.0	4.15
Ag $3d_{3/2}$	373.34	374.0	4.08

SERS Characterization

The highest Raman peak at 556 cm^{-1} can be attributed to the superimposition effect of the S-S stretching vibration and C-S-S symmetric stretching vibration mode[3]. The weak intensity peak centered at 442 cm^{-1} is assigned to the bending vibration of CH_3NC deformation and C=S stretching vibration[4]. The peaks that appeared around 972 cm^{-1} and 848 cm^{-1} are attributed to the C-S-S asymmetric stretching vibration and CH_3N stretching vibration, respectively. The CH_3 symmetric bending vibration and C-N stretching vibration are represented by the peak located at 1372 cm^{-1}. The experimental detection agrees well with the previous reports proposed by Hussain et al.[5], Wang et al.[6], and Xiong et al.[7], except for some slight discrepancies. Furthermore, a minor peak shifted from 1372 to 1380 cm^{-1} for the solid thiram and the tested sample. The minor shift might be explained based on the difference in thiram adsorption on the hot spots of the AuNS@Ag substrate.

References

1. M. Susan Punnoose, D. Bijimol and B. Mathew, *Environmental Nanotechnology, Monitoring & Management*, 2021, **16**, 100525.
2. B. D., *X-ray photoelectron spectroscopy (XPS)*, 2005.
3. K. Liu, Y. Jin, Y. Wu and J. Liang, *Food Chemistry*, 2022, **384**, 132223.
4. X. Zheng, Y. Chen, Y. Chen, N. Bi, H. Qi, M. Qin, D. Song, H. Zhang and Y. Tian, *Journal of Raman Spectroscopy*, 2012, **43**, 1374-1380.
5. A. Hussain, D. W. Sun and H. Pu, *Food chemistry*, 2020, **317**, 126429.
6. B. Wang, L. Zhang and X. Zhou, *Spectrochimica acta. Part A, Molecular and biomolecular spectroscopy*, 2014, **121**, 63-69.

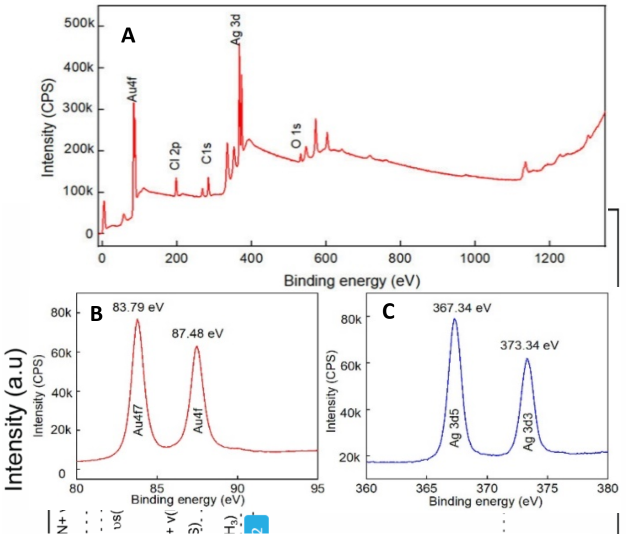

Figure S8. (A) XPS analysis of AuNS@Ag nanoparticles, The high resolution XPS spectrum of (B) Au 4f, and (C) Ag 3d.

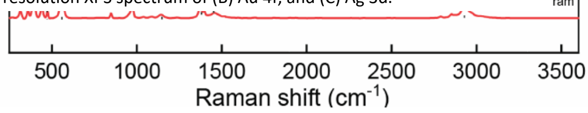

Figure S9. The Raman spectra of thiram (curve red), thiram at the concentration of 0.1 ppm investigated with AuNS (curve blue), AuNS@Ag (curve green), and blank sample prepared with AuNS@Ag nano-substrate (curve black).

7.

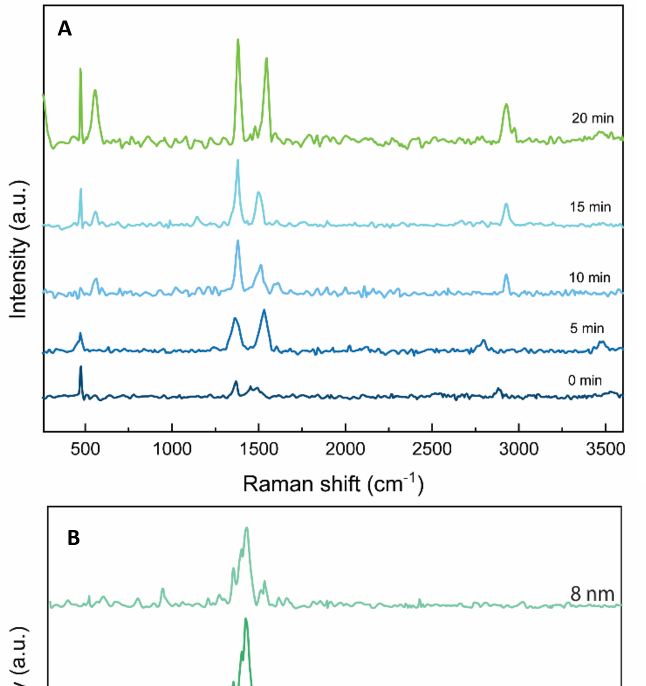

This journal is © The Royal Society of Chemistry 20xx
